# Post–COVID-19 anxiety and its impact on adults’ health in Qatar in 2022

**DOI:** 10.5339/qmj.2026.23

**Published:** 2026-06-04

**Authors:** Nada Adli, Mohamed Bala, Nahlah AlMesbah, Mai Ajabein, Maysa Homaida, Musa Adli Abuhashem, Nada Mohamad, Tajala Fayyaz, Mishkat Awad, Vahe Kehyayan, Abdul Hameed Al-Khenji, Mohamed Ghaith Al-Kuwari, Iheb Bougmiza, Nagah Selim

**Affiliations:** 1 Community and Preventive Medicine Specialist, Preventive Medicine Division, Department of Medicine, Hamad Medical Corporation, Doha, Qatar; 2 Community and Preventive Medicine Department, Primary Health Care Corporation (PHCC), Doha, Qatar; 3 Community and Preventive Medicine Department, Hamad Medical Corporation, Doha, Qatar; 4 Clinical Psychologist, Psychiatry Department, Hamad Medical Corporation, Doha, Qatar; 5 University of Doha for Science and Technology, Doha, Qatar; 6 Senior Consultant of Community and Preventive Medicine, Primary Health Care Corporation (PHCC), Doha, Qatar; 7 College of Medicine, Qatar University, Doha, Qatar; 8 Professor of Community Medicine, Sousse University, Tunisia; 9 Professor of Public Health and Preventive Medicine, Cairo University, Egypt

**Keywords:** Anxiety, post–COVID-19 syndrome, adults, Qatar

## Abstract

**Background:**

Post–COVID-19 syndrome (PCS) is an emerging public health issue characterized by persistent symptoms, including anxiety. Anxiety is the most frequent mental health persistent symptom among patients with PCS, with global prevalence ranging from 6.5% to 63%. It negatively impacts people’s functioning, productivity, and daily routine beyond recovery. This study aimed to examine the prevalence, determinants, and health impact of anxiety symptoms among adults with post–COVID-19 in Qatar during 2022.

**Methods:**

A cross-sectional study was conducted in Qatar between January and July 2022, utilizing data from the Primary Health Care Corporation’s Business Intelligence Unit. Out of 368 eligible individuals, 159 adults with confirmed PCS lasting more than 12 weeks were randomly selected and interviewed by telephone after providing consent. Anxiety levels were assessed using the Generalized Anxiety Disorder-7 (GAD-7) scale.

**Results:**

Out of 159, the prevalence of anxiety symptoms was 50.3% (*n* = 80) based on GAD-7. Most of the participants were female, Qatari, married, and unemployed. Hospitalization and functional impairment were also higher in the anxiety group compared to those without anxiety. Depression, disability, and cognitive dysfunction were strongly associated with the post–COVID-19 anxiety group compared to the non-anxiety group, with P < 0.05.

**Conclusion:**

Post–COVID-19 anxiety is prevalent among PCS patients in Qatar and linked to worse mental and physical health outcomes. Routine screening and integrated care approaches are essential to support recovery.

## 1. INTRODUCTION

COVID-19, first identified in Wuhan in December 2019, was declared a pandemic on March 11, 2020.^1^ Beyond its physical impact, the pandemic significantly affected mental health due to widespread isolation and disruption.^2^ A meta-analysis published in 2020 reported a pooled prevalence of anxiety during the COVID-19 pandemic of 47% (95% CI, 37%–57%; I² = 97A).^3^ Consistently, a systematic review by the COVID-19 Mental Disorders Collaborators in 2021 revealed a 25.6% increase (95% CI, 23.3%–28.0%) in the global prevalence of anxiety compared to pre-pandemic levels.^4^

While many people recovered from COVID-19, a significant proportion developed persistent symptoms over 12 weeks, known as post–COVID-19 syndrome (PCS) or long COVID-19.^5^ PCS affects individuals regardless of age or initial severity of illness, with prevalence estimates ranging from 5% to 80% based on geographic and socio-economic factors.^6^

In the United Kingdom, 100,000 people are estimated to have long COVID, with 66% reporting impaired daily functioning.^7^ One study found 86% of 134 patients had symptoms 113 days post-discharge.^8^ Common symptoms include fatigue (37%), brain fog (32%), sleep issues (31%), memory problems (28%), and anxiety (23%).^9^

Anxiety is the most common psychological symptom after COVID-19, with prevalence ranging from 6.5% to 63%, followed by depression (4%–31%).^10^^,^^11^ Females are 2.2 to 2.5 times more likely to experience post–COVID-19 anxiety.^8^^,^^10^^,^^12^ Anxiety is strongly linked to fatigue, even without prior mental illness,^13^ and together with cognitive and sensory issues, contributes significantly to functional disability.^14^^,^^15^

Anxiety and depression often co-occur, with overlapping rates up to 80% during the pandemic^16^^,^^17^^,^^18^ indicating shared causes. While cognitive symptoms may improve, anxiety, depression, fatigue, and post-traumatic stress disorder (PTSD) can persist beyond a year.^19^

During the COVID-19 pandemic, Qatar implemented a comprehensive and coordinated national response through a multi-agency incident command structure aligned with World Health Organization (WHO) recommendations. Early interventions included travel restrictions, school closures, suspension of mass gatherings, and the rapid conversion of non-essential facilities into isolation and quarantine centers.^20^^,^^21^ Given Qatar’s unique demographic composition, particularly the large population of expatriate workers residing in high-density accommodations, temporary large-capacity isolation facilities were rapidly established to house asymptomatic COVID-19–positive individuals,^22^ allowing effective physical distancing and reducing community transmission. Hospital capacity was promptly expanded through collaboration between public and private sectors, and a nationwide digital application was launched to facilitate contact tracing and quarantine monitoring. In parallel, early testing strategies were expanded by introducing drive-through testing clinics, enabling efficient screening of returning travelers and their contacts while minimizing hospital congestion and person-to-person exposure.^23^ These measures were further informed by national public health control strategies that recognized the role of asymptomatic and pre-symptomatic transmission, population mobility, and high-risk living and working environments in Qatar.^24^ Testing and treatment were provided free of charge through the state health system, contributing to a relatively low case-fatality rate.^25^ The Primary Health Care Corporation (PHCC), Qatar’s leading primary care provider serving over 1.6 million residents across 28 health centers, played a pivotal role by offering COVID-19 testing at all facilities, with positive cases promptly reported to PHCC’s Business Health Intelligence Unit and communicated to the Ministry of Public Health.^26^ Despite these efforts, research on post–COVID-19 anxiety in Qatar remains limited. To address this gap, the present study aims to determine the prevalence of post–COVID-19 anxiety, characterize the spectrum of persistent symptoms, and assess their impact on the physical, psychological, and cognitive functioning of affected individuals.

## 2. MATERIALS AND METHODS

### 2.1 Study design and setting

This cross-sectional study, which was conducted in Qatar as part of a larger published project,^27^ involved the analysis of confirmed COVID-19 cases from January to July 2022. Data was sourced from the Business Intelligent Unit (BIU) of PHCC,^26^ which maintains comprehensive electronic medical records across all PHCC-affiliated healthcare centers. Serving a population exceeding 1.6 million through 31 centers, PHCC was integral to the national COVID-19 response, facilitating widespread testing and reporting positive cases to the Ministry of Public Health (MOPH).^26^

### 2.2 Sample size and sampling method

This study was part of a larger project^27^ that initially included 368 patients with confirmed COVID-19 infection, identified through the BIU unit of the PHCC. From this cohort, a subset of 159 participants was selected for the present sub-analysis. These participants were adults aged 18 years and older who identified as having PCS (experienced persistent symptoms lasting more than 12 weeks). Eligible participants were Arabic- or English-speaking, while those with pre-existing psychological disorders, cognitive impairment, or disability were excluded.

### 2.3 Questionnaire development and validation process

The questionnaire was developed and constructed by the authors following an extensive literature review. It comprised 12 questions about socio-demographic and health-related characteristics. These include information about age, gender, nationality, marital status, educational level, employment status, perceived monthly income, and Household Crowding Index (HCI).^28^ Additionally, we included questions about co-morbidities, height, weight, hospital admissions, and the presence of other persistent symptoms after recovery.

The face validity of the questionnaire was assessed and confirmed through expert consultation with community and preventive medicine physicians and specialists in the relevant field. To ensure translation validity, the English version was translated into Arabic by two native Arabic speakers and then back-translated into English to check for consistency. All authors reviewed and agreed upon the final version. Subsequently, the questionnaire underwent piloting on a convenience sample of 35 adults aged 18 years and above to assess its clarity, comprehensibility, and appropriateness.

### 2.4 Data collection tools and variables

Data were collected through structured telephone interviews conducted by trained physicians fluent in both Arabic and English, using a bilingual questionnaire to ensure clarity and comfort. Each interview lasted approximately 40 to 45 minutes and was scheduled at the participant’s preferred time to facilitate participation. When immediate participation was not possible, an alternative time was arranged to ensure full engagement. Each participant was interviewed once, and all required data were obtained during that single session in their preferred language.

Participation in the study was voluntary, and participants had the right to withdraw at any time. Only those who agreed to participate were included.

Before participation, individuals were informed about the study objectives and provided their consent. The data collection tools included: (1) A data extraction sheet containing participants’ telephone numbers and the dates of their last confirmed COVID-19 infection. (2) A structured questionnaire capturing socio-demographic characteristics and health-related information.

### 2.5 Dependent variables

The primary outcome variable was the prevalence of post–COVID-19 anxiety, evaluated using the Generalized Anxiety Disorder-7 (GAD-7) scale.^29^ The GAD-7 is a validated, reliable instrument widely employed in both clinical and research settings to screen for anxiety and quantify its severity. It demonstrates robust psychometric properties, with a sensitivity of 0.77 and specificity of 0.82. The scale comprises seven items scored on a 0 to 3 Likert scale, yielding a total score range of 0 to 21, with higher scores indicating greater anxiety severity.^29^ Participants were subsequently classified into two groups, those with post–COVID-19 anxiety and those without, based on established GAD-7 cut-off values.

### 2.6 Independent variables

The data collection tool was used to gather information about participants’ sociodemographic characteristics, including age, gender, nationality, marital status, educational level, employment status, and perceived monthly income. Participants’ perceptions of their monthly income were assessed by a Likert scale and initially classified into four levels: “More Than Enough, Enough, Just Barely Enough, and Not Enough at All”. The HCI was measured by calculating the total number of co-residents per household, excluding newborn infants, divided by the total number of rooms, excluding the kitchen and bathroom. It was then re-grouped into three categories: (<1, 1–2, and > 2). A higher HCI indicates lower Socio-Economic Status (SES).^28^

Regarding health-related characteristics, the presence of co-morbidities was assessed by two categories: yes or no, and the type of co-morbidities, such as hypertension, diabetes mellitus, and asthma, etc. Additionally, information about height and weight was collected. The body mass index (BMI) was calculated for all participants and dichotomized into “obese” (BMI ≥ 30 kg/m^2^) and “not obese” (BMI < 30 kg/m^2^) groups.^30^ Hospital admissions (yes or no) were also assessed for all participants.

Additionally, other health problems were assessed using standardized and validated tools. Fatigue was measured with the Fatigue Severity Scale (FSS), a reliable instrument (Cronbach’s α = 0.928) consisting of nine items rated on a 7-point Likert scale (1 = “strongly disagree” to 7 = “strongly agree”); a total score ≥ 36 indicates severe fatigue.^31^^,^^32^ Dyspnea was evaluated using the Medical Research Council (MRC) Breathlessness Scale, a single-item, five-grade tool widely validated across populations,^33^ with higher scores indicating greater severity. Depression was assessed with the Patient Health Questionnaire-9 (PHQ-9), a valid and reliable tool (sensitivity and specificity = 88%) using a 4-point Likert scale (0 = “not at all” to 3 = “nearly every day”), with total scores categorized by severity.^34^ Cognitive dysfunction was measured using the Neuro-QoL cognitive function scale, a validated instrument (Cronbach’s α ≥ 0.94) comprising eight items rated from 1 (“severe problem”) to 5 (“no problem”); total scores < 30 indicate severe dysfunction.^35^ Finally, disability was assessed with the World Health Organization Disability Assessment Schedule 2.0 (WHODAS 2.0), validated in Arabic and English (Cronbach’s α = 0.74). This 5-point Likert scale (1 = “none” to 5 = “cannot do”) defines higher scores as indicating greater disability.^36^^,^^37^^,^^38^

### 2.7 Ethical consideration

This study was approved by the PHCC Ethical Committee under protocol PHCC/DCR/2022/06/31. Verbal consent was obtained from each participant before the interview. The study was conducted in full conformance with the principles of the “Declaration of Helsinki” and Good Clinical Practice (GCP).

### 2.8 Analysis

Data was analyzed using SPSS v. 25. Descriptive statistics summarized participants’ characteristics, and normality was assessed using the Kolmogorov-Smirnov test. Chi-squared tests examined associations with post–COVID-19, with significance set at P ≤ 0.05.

## 3. RESULTS

### 3.1 Levels of post–COVID-19 anxiety

**Figure 1.**
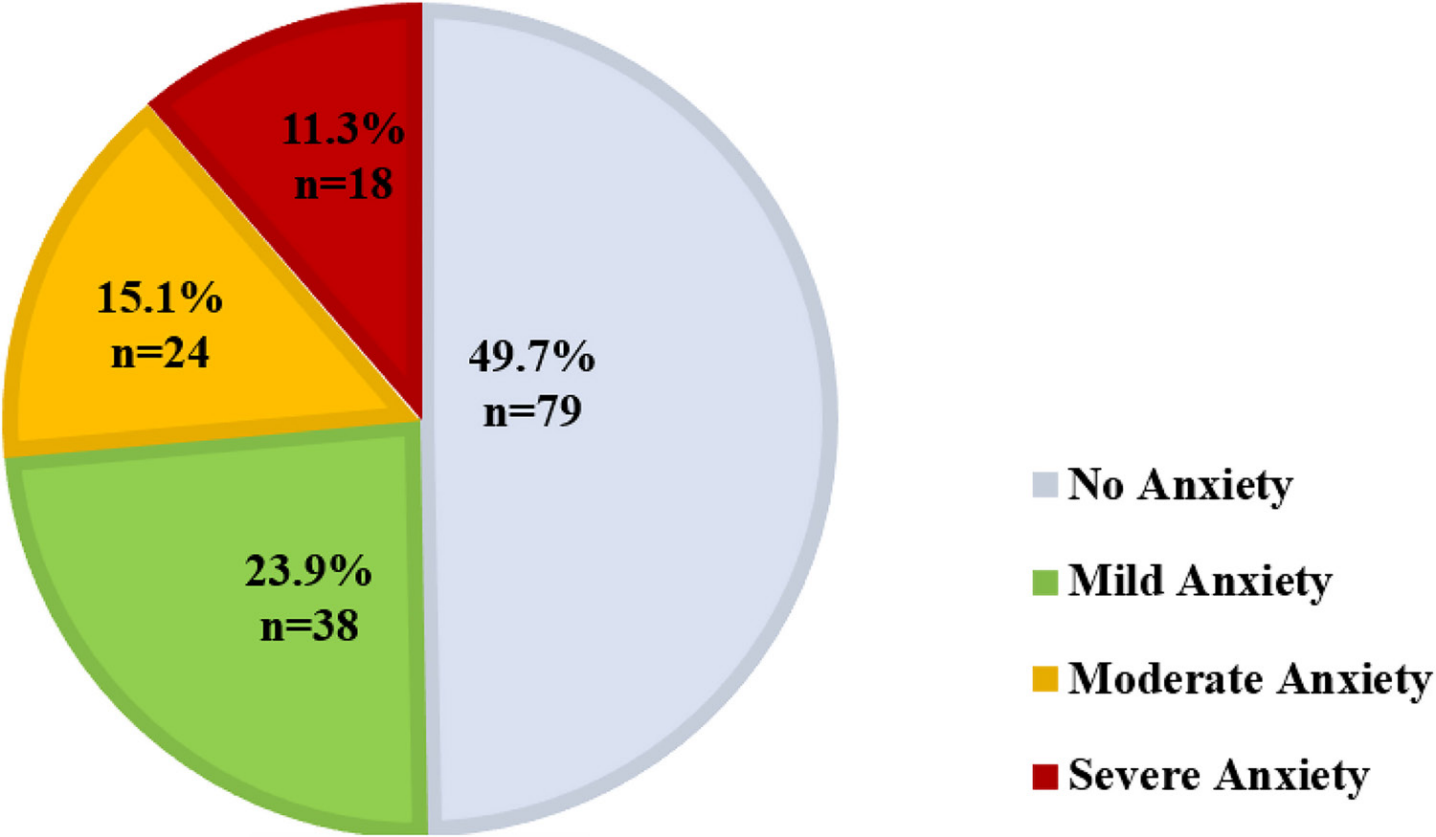
Prevalence of anxiety symptoms among primary health care patients in Qatar with post–COVID-19 syndrome during 2022 (*N* = 159).

Among 159 participants, 50.3% (*n* = 80) reported anxiety symptoms based on the GAD-7 scale. Severity was classified as no anxiety (49.7%), mild (23.9%), moderate (15.1%), and severe (11.3%; Figure 1). Participants were grouped into post–COVID-19 anxiety and non-anxiety categories accordingly.

**Figure 2.**
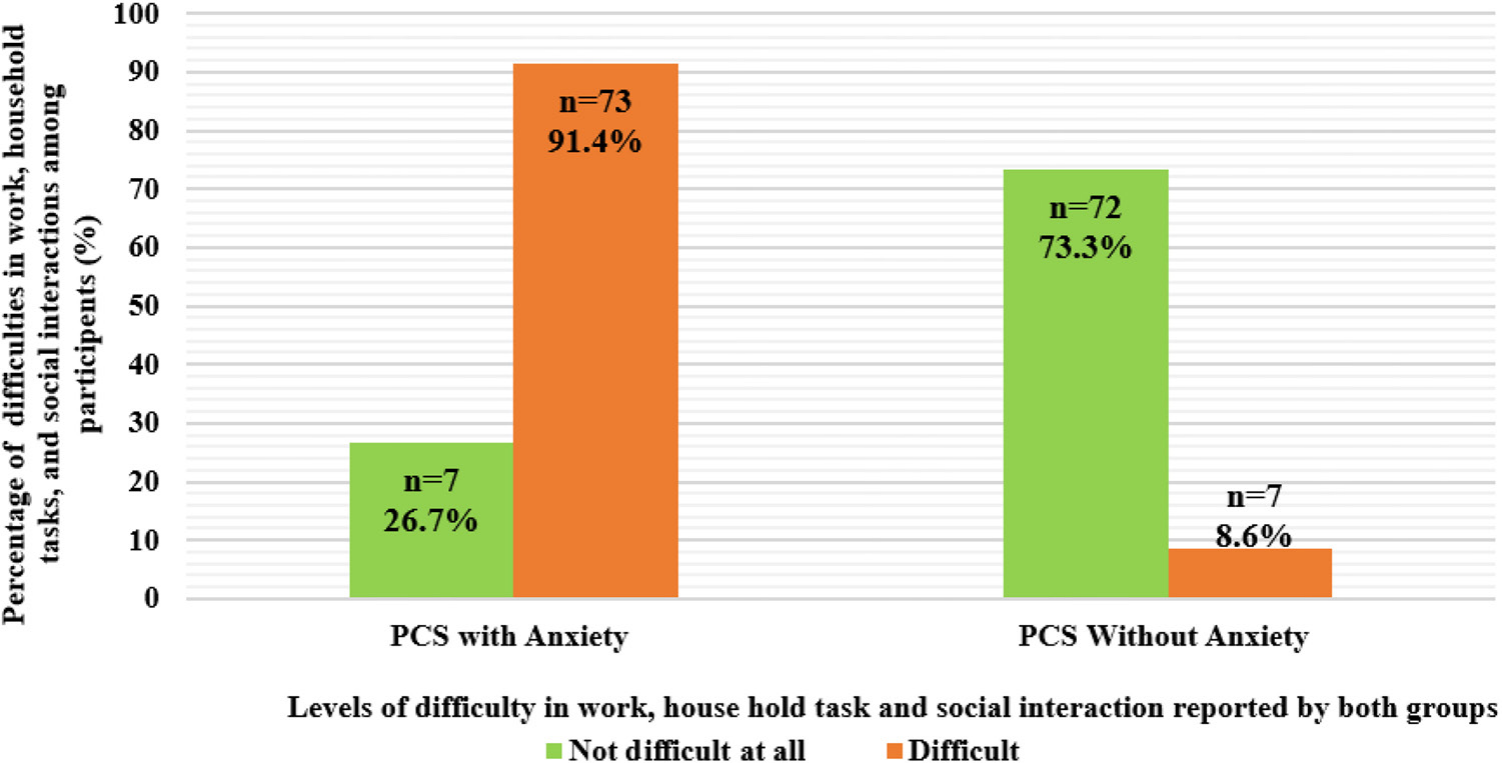
Frequency distribution of perceived difficulty level in work, household, and social activities among post–COVID-19 patients with and without anxiety in Qatar, 2022 (*N* = 159).

PCS patients with anxiety reported significantly greater difficulties in work, household tasks, and social interactions (91.4%, *n* = 73) compared to those without anxiety (8.6%, *n* = 7), with a P value ≤ 0.05, as shown in Figure 2.

Table 1 shows the relationship between socio-demographic and health-related factors and anxiety in PCS. No statistically significant differences were observed between the two groups regarding age, Gender, nationality, marital status, educational attainment, employment status, perceived monthly income, HCI, or medical history.

**Figure 3.**
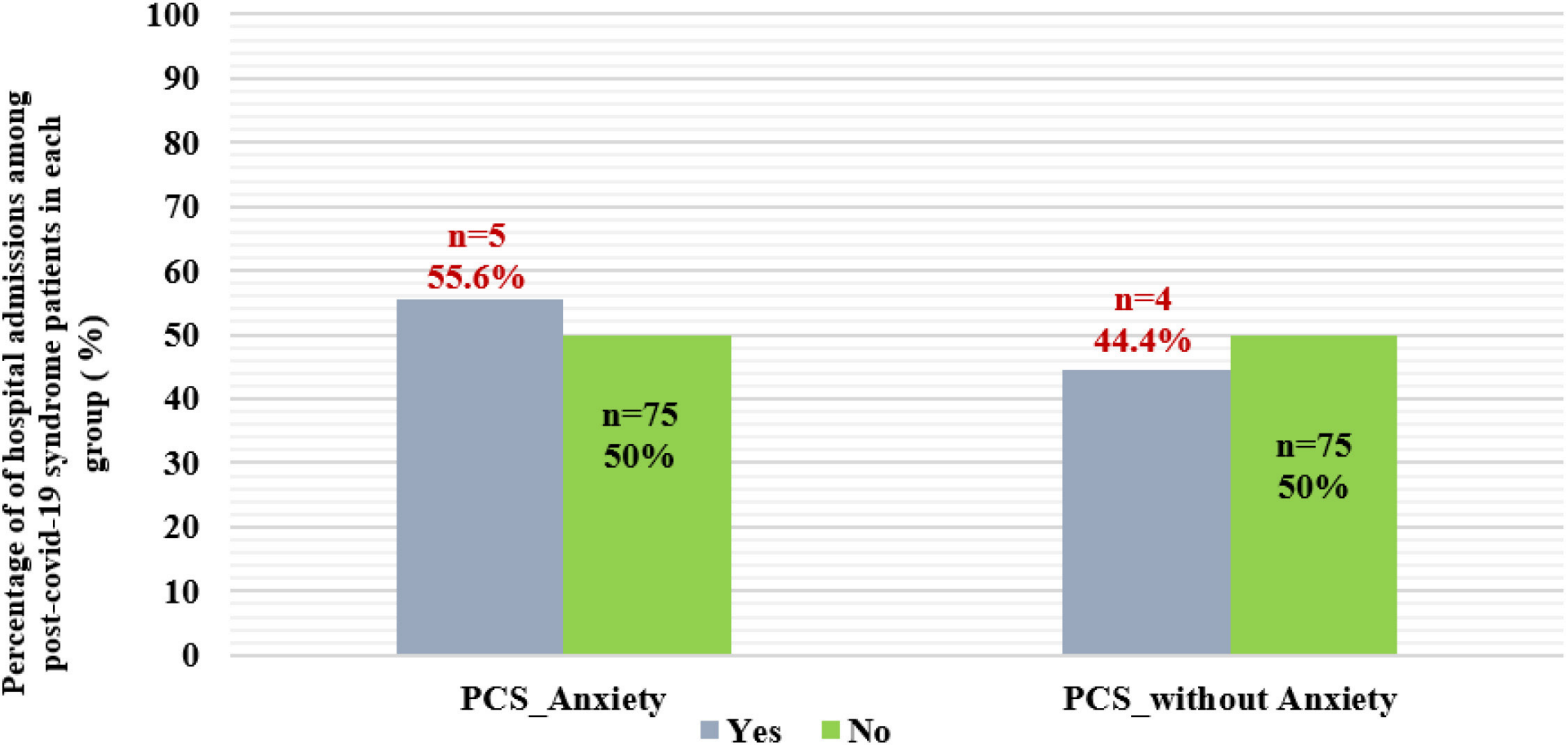
Frequency distribution of hospital admissions among post–COVID-19 syndrome patients with and without anxiety in Qatar during 2022 (*N* = 159).

As shown in Figure 3, hospital admissions were higher among the post–COVID-19 anxiety group compared to the post–COVID-19 non-anxiety group (55.6% vs. 44.4%), however, this difference was not statistically significant (P = 0.10).

Participants with post–COVID-19 anxiety reported significantly higher rates of persistent symptoms, particularly fatigue, forgetfulness, mood changes, depression, and general weakness, compared to those without anxiety (Table 2).

As shown in Table 3, post–COVID-19 anxiety was significantly associated with greater cognitive dysfunction, higher depression severity, increased disability, and poorer perceived health (P = 0.024, 0.001, and 0.033, respectively).

## 4. DISCUSSION

This study aimed to determine the prevalence of anxiety symptoms among adults with PCS and their impact on adult health in Qatar in 2022.

Psychological problems, including anxiety, have been reported in several studies as negative consequences experienced by PCS following their recovery from COVID-19 infection.^39^^,^^40^ Moreover, a scoping review by Shanbehzadeh et al. found that anxiety was the most frequently reported mental health symptom, with PCS prevalence ranging from 6.5% to 63% at follow-up times extending beyond one month after the initial COVID-19 infection.^10^ Depression is also frequently reported, with prevalence rates between 4% and 31%.^10^^,^^11^ A meta-analysis by Premraj et al. including studies from multiple countries, reported that fatigue affected 37% of patients, brain fog 32%, sleep disturbances 31%, memory issues 28%, and anxiety 23% among post–COVID-19 patients.^9^ Furthermore, a meta-analysis by Shanbehzadeh et al. demonstrated that the prevalence of anxiety increased over time, with higher rates observed at long-term follow-up, 6 months or more after infection, compared with mid-term follow-up, 3 to 6 months after infection.^10^ In the present study, we used the GAD-7 scale tool to investigate the severity of anxiety among our population. Findings indicated that anxiety is common among adults with PCS in Qatar. Notably, there were no significant associations between anxiety and demographic or clinical characteristics. However, fatigue, forgetfulness, mood alteration, depressive symptoms, and general weakness were the symptoms most associated with anxiety.

These findings are in line with the current evidence in the literature. Several studies have investigated the prevalence of anxiety in patients with PCS. According to Sykes et al. anxiety was found to be common (47%) among 134 patients in the United Kingdom at a median of 113 days (range = 46–167) post-hospital discharge.^8^ Similarly, Badinlou et al. showed a higher prevalence of anxiety (55.5%) among 507 adult patients with a history of probable or confirmed COVID-19 diagnosis in Sweden.^15^ However, these findings were self-reported by the participants in their study and were not assessed by a standardized tool.

In contrast, some studies have reported the lower prevalence of anxiety among post–COVID-19 patients. For example, a study conducted by Menges et al. involving 431 participants found that 32% (*n* = 135) reported anxiety symptoms.^41^ Similarly, a study from China showed a relatively low level of anxiety (22.46%).^42^ These discrepancies between our study and others may be partly explained by differences in selection criteria, such as age, gender, and disease severity. Another contributing factor could be variations in follow-up duration and data collection methods, such as the use of online follow-up platforms in the study by Ismael et al.^42^ Additionally, differences in the instruments used to measure anxiety may also influence study outcomes.

Moreover, a study conducted by Huarcaya-Victoria et al. found an anxiety prevalence of 31.1% among 318 patients after hospital discharge in Peru (mean follow-up = 102 days).^43^ This lower prevalence may be explained by the relatively small proportion of female participants (38.7%).

In our study, 68% of participants were female, and female gender was nearly statistically significantly associated with anxiety. This observation is consistent with a recent systematic review by Zakia et al. and several other studies,^44^^,^^45^^,^^46^^,^^47^ which identified female gender as a major risk factor for developing anxiety in PCS. Similarly, Shanbehzadeh et al. in Iran, reported that females were 2.2 to 2.5 times more likely than males to experience post–COVID-19 mental health disturbances, including anxiety.^8^^,^^10^^,^^12^

In addition, recent evidence suggests that obesity and mental health disorders, including depression and anxiety, have a bidirectional relationship and share overlapping biological, behavioral, and psychosocial pathways, such as chronic inflammation, metabolic dysregulation, and lifestyle-related factors.^48^^,^^49^ However, in our study, obesity was not found to be statistically significantly associated with anxiety (Table 1). This may be partly attributable to the high prevalence of non-communicable diseases and sedentary lifestyles in Qatar’s population, which could limit the ability to detect clear associations and make direct comparisons inconclusive.^50^

Furthermore, our study revealed that anxiety was significantly associated with somatic symptoms such as fatigue (55.8% vs. 44.2%; P-value = 0.0015) and general weakness (63.5% vs. 36.5%; P-value = 0.007; Table 2), suggesting that these symptoms could either contribute to or be exacerbated by anxiety. There is also evidence that the pathophysiology of anxiety in PCS could be explained by residual inflammatory processes that affect the brain, potentially leading to manifestations^45^^,^^46^^,^^47^ of other psychiatric symptoms like depression and mood alteration. Our findings support this, as anxiety was significantly associated with the severity of depressive symptoms (88.9% vs. 11.1%; P-value = 0.001) and mood alterations (73.6% vs. 26.4%; P-value = 0.000; Tables 2 and 3). Furthermore, anxiety was significantly associated with worse disability scores (73.0% vs. 27.0%; P-value = 0.001) and poorer perception of health (84.6% vs. 15.4%; P-value = 0.03; Table 3). These findings are supported by international evidence highlighting the interplay between fatigue, cognitive dysfunction, and psychological outcomes in PCS. Townsend et al. in Ireland, reported that post–COVID-19 fatigue at a median of 166 days post-infection was significantly associated with anxiety, even in individuals without a prior history of anxiety (P < 0.001).^13^ Cacciatore et al. in Italy, found that cognitive dysfunction, anxiety, fatigue, and hyposmia/hypogeusia collectively explained 28.8% of the variance in WHODAS-II disability scores at 3.5 months post-infection, indicating these symptoms as key contributors to functional impairment.^14^ Similarly, Badinlou et al. in Sweden, demonstrated significant positive correlations between post–COVID-19 impairments, fatigue, and psychological outcomes, with fatigue and functional limitations accounting for 12% of the variance in anxiety symptoms.^15^ Latronico et al. in Italy, further showed that although cognitive impairment may improve over time, fatigue, depression, anxiety, insomnia, and PTSD symptoms often persist for at least 1 year after infection.^19^

The comorbidity of anxiety and depression in PCS has been well documented. In the United States, Judd et al. reported that 67% of patients with generalized anxiety disorder also met criteria for unipolar depressive disorder,^16^ while Stein and Uhde observed that 19.2% of patients in primary care presented with co-occurring anxiety and depression.^17^ More recently, Long et al. in a cross-national study across eight countries, found that 80% of individuals who met criteria for anxiety also fulfilled criteria for depression during the COVID-19 pandemic.^18^

Regarding the longitudinal trajectory of post–COVID-19 neuropsychiatric symptoms, Latronico et al. conducted a 1-year follow-up of an Italian cohort and demonstrated a significant reduction in cognitive impairment; however, fatigue, depression, anxiety, insomnia, and PTSD symptoms persisted without notable resolution.^19^ Collectively, these studies underscore the complex interplay between somatic, cognitive, and psychological symptoms in PCS, highlighting the need for integrated management strategies to address both mental health and functional impairment.

### 4.1 Study strengths and challenges

Our study had several strengths. For instance, the WHO’s case definition for PCS was adopted. Furthermore, only laboratory-confirmed COVID-19 infections were included. This may reduce the selection bias that has been shown in other studies where probable self-reported COVID-19 infections were used. Additionally, contrary to other studies, which only investigated hospitalized patients, our study included both hospitalized and non-hospitalized patients, which means we covered the full spectrum of mild, moderate, and severe COVID-19-infected individuals. This may have reduced selection bias as well.

Finally, we used standardized assessment tools, such as the GAD-7 scale, PHQ-9, FSS, MRC-Dyspnea scale, Neuro-QoL, and WHODAS-II. We also had well-trained data collectors, all of whom were qualified physicians fluent in both Arabic and English. They received comprehensive training on confirming participants’ eligibility, explaining study objectives, obtaining consent, and administering the questionnaire through structured telephone interviews. In addition, they were trained to ensure participants’ confidentiality and emphasize their right to withdraw at any time. This rigorous training process helped maintain high data quality and accuracy, thereby enhancing the overall reliability and credibility of the study findings.

A cross-sectional telephone design may introduce recall and selection bias, as non-respondents may differ in key characteristics or symptom reporting. Moreover, the use of GAD-7 alone may not capture the full spectrum of anxiety-related conditions such as panic disorder, PTSD, or functional impairment in post–COVID-19 patients. Another limitation of this study is that only face validity was assessed for the Arabic versions of the tools, and the validation of the original English versions, including sensitivity and specificity, is not directly transferable.

### 4.2 Conclusion and recommendations

In Qatar, nearly half of adults with PCS experienced anxiety, which was associated with higher hospital admissions compared to those without anxiety. Patients with anxiety also reported greater difficulties in work, household tasks, and social interactions, alongside higher levels of depression, disability, cognitive dysfunction, and poorer perceived health. These findings suggest that anxiety may serve as an indicator of PCS severity and underscore the importance of routine anxiety screening to ensure timely rehabilitation and psychosocial support. Further research is needed to better understand the mental health impact of PCS within the population in Qatar.

## ACKNOWLEDGEMENT

The authors would like to sincerely thank the Qatar Medical Journal for its support in facilitating the publication process.

## FUNDING

This research did not receive any specific grant from funding agencies in the public, commercial, or not-for-profit sectors.

## CONFLICT OF INTEREST

The authors declare that the research was conducted in the absence of any commercial or financial relationships that could be construed as potential conflicts of interest.

## ETHICAL APPROVAL

This study was approved by the PHCC Ethical Committee (PHCCIRB) under protocol number (PHCC/DCR/2022/06/31). Verbal consent was obtained from each participant before the interview. The study was conducted in full conformance with the principles of the “Declaration of Helsinki” and GCP.

## AUTHOR CONTRIBUTIONS

NA: Conceptualization, methodology, investigation, formal analysis, data curation, project administration, writing–original draft, and writing–review and editing. MB and NAM: Conceptualization, methodology, writing–original draft, and writing–review and editing. MA, MH, MAA, NM, TF, and MA: Investigation, data curation, writing–review and editing. VK, AHA-K, MGA-K, and IB: Methodology, supervision, writing–critical review, and editing. NS: Methodology, formal analysis, data curation, supervision, review, and editing.

## DATA AVAILABILITY STATEMENT

The data sets generated during and/or analyzed during the current study are available from the corresponding author on reasonable request.

## DISCLOSURE OF AI USE

The authors also declare that artificial intelligence (AI) tools were used solely for language editing and formatting of the manuscript. No AI tools were used for data analysis, interpretation of results, or generation of scientific content, and all intellectual content and conclusions are entirely the responsibility of the authors.

## Figures and Tables

**Table 1.** Relationship between socio-demographic and health-related characteristics among adults with PCS and anxiety (*N* = 159).

	Post–COVID-19 anxiety		
		Yes (*n* = 80)	No (*n* = 79)	
	Variable	*n* (%)	*n* (%)	*P* value
**Age, years**			
18–24	6(60.0)	4 (40.0)	0.867
25–39	52 (50.0)	52 (50.0)	
≥40	22 (48.9)	22 (51.1)	
Mean age (SD), years	36.3 ± 9.9	36.8 ± 9.4	0.554
**Gender**			
Male	20 (39.2)	31 (60.8)	0.054
Female	60 (55.6)	48 (44.4)	
**Nationality**			
Qatari	10 (62.5)	6 (37.5)	0.304
Non-Qatari	70 (49.0)	73 (51.0)	
**Marital status**			
Single	19 (45.2)	23 (54.8)	0.443
Married	61 (52.1)	56 (47.9)	
**Education status**			
Secondary and less	12 (46.2)	14 (53.8)	0.643
University/higher education	68 (51.1)	65 (48.9)	
**Employment status**			
Employed	57 (46.7)	65 (53.3)	0.100
Not-employed	23 (62.2)	14 (37.8)	
**Perceived monthly income**			
Not enough	13 (39.4)	20 (60.6)	0.354
Enough	62 (53.4)	54 (46.6)	
More than enough	5 (50.0)	5 (50.0)	
**Household Crowding Index**			
High socioeconomic status	13 (54.2)	11 (45.8)	0.797
Middle socioeconomic status	61 (50.4)	60 (49.6)	
Low socioeconomic status	6 (42.9)	8 (57.1)	
**Medical history**			
**Diabetes mellites**
Yes	6 (60.0)	4 (40.0)	0.746
No	74 (49.7)	75 (50.3)	
**Hypertension**			
Yes	9 (69.2)	4 (30.8)	0.155
No	71 (48.6)	75 (51.4)	
**Cardiovascular diseases**			
Yes	2 (50.0)	2 (50.0)	1.000
No	78 (50.3)	77 (49.1)	
**Asthma**			
Yes	4 (44.4)	5 (55.6)	0.746
No	76 (50.7)	74 (49.3)	
**Thyroid disease**			
Yes	3 (42.9)	4 (57.1)	0.719
No	77 (50.7)	75 (49.3)	
**Obese**			
Yes	57 (47.5)	63 (52.5)	0.213
No	23 (59.0)	16 (41.0)	

*n* = Frequency; % = Percentage.

*P ≤ 0.05; **P ≤ 0.01; ***P ≤ 0.001.

**Table 2.** The association between persistent post–COVID-19 symptoms and anxiety among adults with post–COVID-19 syndrome in Qatar, 2022 (*N* = 159).

	Post–COVID-19 anxiety		
	Yes (*n* = 80)	No (*n* = 79)	
	*n* (%)	*n* (%)	*P* value
**Fatigue**			
Yes	67 (55.8)	53 (44.2)	0.015**
No	13 (33.3)	26 (66.7)	
**Sleep disturbance**			
Yes	9 (52.9)	8 (47.1)	0.819
No	71 (50.0)	71 (50.0)	
**Low concentration**			
Yes	15 (50.0)	15 (50.0)	0.969
No	65 (50.4)	64 (49.6)	
**Forgetfulness**			
Yes	47 (63.5)	27 (36.5)	0.002**
No	33 (38.8)	52 (61.2)	
**Memory loss**			
Yes	7 (70.0)	3 (30.0)	0.328
No	73 (49.0)	76 (51.0)	
**Confusion**			1.000
Yes	2 (50.0)	2 (50.0)	
No	78 (50.3)	77 (49.7)	
**Mood alteration**			0.000***
Yes	53 (73.6)	19 (26.4)	
No	27 (31.0)	60 (69.0)	
**Depressive symptoms**			
Yes	31 (72.1)	12 (27.9)	0.001***
No	49 (42.2)	67 (57.8)	
**General weakness**			0.007**
Yes	40 (63.5)	23 (36.5)	
No	40 (41.7)	56 (58.3)	
**Hair loss**			
Yes	19 (54.3)	16 (45.7)	0.595
No	61 (49.2)	63 (50.8)	
**Menstrual cycle disturbance (female = 108)**			
Yes	16 (51.6)	15 (48.4)	0.601
No	44 (57.1)	33 (42.9)	

SD = Standard deviation; *n* = Frequency; % = Percentage; CI = Confidence interval.

*P ≤ 0.05; **P ≤ 0.01; ***P ≤ 0.001.

Bold values indicate statistical significance (≤ 0.05).

**Table 3.** The relationship between the severity of some persistent symptoms among adults with PCS and anxiety (*N* = 159).

	Post–COVID-19 anxiety		
	Yes (*n* = 80)	No (*n* = 79)	
	*n* (%)	*n* (%)	*P* value
**Fatigue Severity Scale**			
Yes	40 (54.8)	33 (45.2)	0.298
No	40 (46.5)	46 (53.5)	
**Dyspnea Scale**			
Mild	46 (46.5)	53 (53.5)	0.431
Moderate	29 (55.8)	23 (44.2)	
Severe	5 (62.5)	3 (37.5)	
**Cognition dysfunction**			
None	57 (44.9)	70 (55.1)	0.024*
Mild	11 (73.3)	4 (26.7)	
Moderate/severe	12 (70.6)	5 (29.4)	
**Depression severity**			
None–minimum	34 (37.0)	58 (63.0)	
Mild	17 (58.6)	12 (41.4)	
Moderate	14 (77.8)	4 (22.2)	0.001**
Moderately severe	7 (63.6)	4 (36.4)	
Severe	8 (88.9)	1 (11.1)	
**Disability**			
No disability	17 (32.1)	36 (67.9)	
Mild disability	16 (43.2)	21 (56.8)	0.001**
Moderate disability	20 (62.5)	12 (37.5)	
Severe disability	27 (73.0)	10 (27.0)	
Overall health in the past 30 days			
Good	49 (46.2)	57 (53.8)	0.033*
Moderate	20 (50.0)	20 (50.0)	
Bad	11 (84.6)	2 (15.4)	

*n* = Frequency; % = Percentage; CI = Confidence interval.

*P ≤ 0.05; **P ≤ 0.01; ***P ≤ 0.001.

Bold values indicate statistical significance (≤ 0.05).
